# GATExplorer: Genomic and Transcriptomic Explorer; mapping expression probes to gene loci, transcripts, exons and ncRNAs

**DOI:** 10.1186/1471-2105-11-221

**Published:** 2010-04-29

**Authors:** Alberto Risueño, Celia Fontanillo, Marcel E Dinger, Javier De Las Rivas

**Affiliations:** 1 Bioinformatics and Functional Genomics Research Group, Cancer Research Center (CiC-IBMCC, CSIC/USAL), Salamanca, Spain; 2 Institute for Molecular Bioscience, University of Queensland, Brisbane, Australia

## Abstract

**Background:**

Genome-wide expression studies have developed exponentially in recent years as a result of extensive use of microarray technology. However, expression signals are typically calculated using the assignment of "probesets" to genes, without addressing the problem of "gene" definition or proper consideration of the location of the measuring probes in the context of the currently known genomes and transcriptomes. Moreover, as our knowledge of metazoan genomes improves, the number of both protein-coding and noncoding genes, as well as their associated isoforms, continues to increase. Consequently, there is a need for new databases that combine genomic and transcriptomic information and provide updated mapping of expression probes to current genomic annotations.

**Results:**

GATExplorer (Genomic and Transcriptomic Explorer) is a database and web platform that integrates a gene loci browser with nucleotide level mappings of oligo probes from expression microarrays. It allows interactive exploration of gene loci, transcripts and exons of human, mouse and rat genomes, and shows the specific location of all mappable *Affymetrix *microarray probes and their respective expression levels in a broad set of biological samples. The web site allows visualization of probes in their genomic context together with any associated protein-coding or noncoding transcripts. In the case of all-exon arrays, this provides a means by which the expression of the individual exons within a gene can be compared, thereby facilitating the identification and analysis of alternatively spliced exons. The application integrates data from four major source databases: *Ensembl*, *RNAdb, Affymetrix *and *GeneAtlas*; and it provides the users with a series of files and packages (R CDFs) to analyze particular query expression datasets. The maps cover both the widely used *Affymetrix GeneChip *microarrays based on 3' expression (e.g. human HG U133 series) and the all-exon expression microarrays (Gene 1.0 and Exon 1.0).

**Conclusions:**

GATExplorer is an integrated database that combines genomic/transcriptomic visualization with nucleotide-level probe mapping. By considering expression at the nucleotide level rather than the gene level, it shows that the arrays detect expression signals from entities that most researchers do not contemplate or discriminate. This approach provides the means to undertake a higher resolution analysis of microarray data and potentially extract considerably more detailed and biologically accurate information from existing and future microarray experiments.

## Background

As our knowledge of metazoan genomes and transcriptomes improves, the number of both protein-coding and noncoding transcripts continues to increase [1,2]. To take account of the increasing emphasis on transcriptomics, genomic databases need to be adapted to better accommodate this type of data. Consideration of transcriptomic data necessitates improvements in both the correct mapping of all actively transcribed units and the accurate determination of their expression levels. *Ensembl *maintains and provides visualization of a comprehensive database of all publicly available eukaryotic genome sequences and contains all major biomolecular entities (such as RNAs and proteins) with extensive additional information including mapping of microarray probes [3]. Other databases, such as *RNAdb*, complement *Ensembl *by providing details and annotations of larger collections of non-coding RNAs (ncRNAs) [4]. However, these biological databases do not integrate expression signal data and they do not provide tools to use up-to-date probe mapping with query expression datasets. Finally, databases such as *GEO *include large collections of expression datasets with powerful analysis tools, but they lack microarray probe mapping at nucleotide level and presentation in a genomic context, and instead consider "probesets" as genes [5]. Several recent transcriptomic studies are showing that gene loci are considerably more complex than previously thought, often with networks of overlapping transcripts on both strands [1], emphasizing the importance to examine expression data at the nucleotide level. Nucleotide level mapping facilitates the identification of particular probes that uniquely represent the expression of specific transcripts. It also provides the possibility to discriminate between alternate isoforms of the same gene. Such analyses require unambiguous assignment of the array probes to the functional entities defined in current transcriptomes (i.e. gene loci, transcripts, exons, ncRNAs), including their specific genomic location. The huge number of transcriptomic studies conducted in recent years illustrates the potential demand for improved analytical approaches of microarray data as well as the opportunity to reinterpret existing datasets. To provide a means to analyze microarray data at the nucleotide level in a genomic context we have developed a database and web platform called GATExplorer. The application integrates information from multiple biological sources and includes several bioinformatic tools to allow a novel perspective and interpretation of microarray expression data.

## Construction and content

### Database integrating genomes, transcriptomic entities and expression

To analyze transcriptomic data in a genomic context, GATExplorer integrates five **datasets**: **(i) **the human, mouse and rat genomes (derived from *Ensembl *http://www.ensembl.org); **(ii) **the sequences and IDs of all oligonucleotide probes (perfect match only) from all *Affymetrix *expression microarrays http://www.affymetrix.com for these species; **(iii) ***de novo *mapping data of each array probe to the transcriptome of the corresponding organism, with the genomic coordinates for each locus (including locations on exons, introns and across exon-exon junctions) and identification of any intersecting genes, transcripts and exons; **(iv) **mapping data of unmapped probes to transcripts in *RNAdb *(research.imb.uq.edu.au/RNAdb), a database of ncRNAs of human and mouse; and **(v) **detailed expression data derived from a set of microarrays from different cell types, tissues or organs (*GeneAtlas *GEO ID GSE1133 [6]) calculated at probe- and probeset-level using complete *de novo *mapping.

BLASTN sequence **alignment **was used to map the 25-mer oligo probes of the main *Affymetrix *expression microarrays to the RNA sequences of human, mouse and rat, selecting only complete perfect match alignments. The mapped probes were then placed in the corresponding genome based on the coordinates of the main genomic entities defined by *Ensembl*. The versions of the *genomes assemblies *and the *source databases *in current use are indicated on the website (PROBE MAPPING section, "Genomes ASSEMBLY and Databases VERSION").

Each of the source and newly derived datasets are structured and integrated in a relational SQL database (MySQL), which can be queried and viewed via the website. For a specified gene locus, the web interface presents a hierarchical display of the corresponding genomic entities (chromosome, locus, exons, transcripts and protein domains), together with detailed mapping of array probes and probesets and their signal in a set of sample arrays. This data is presented as follows: **(i) **Description; **(ii) **Chromosome global view (chr [chr number]); **(iii) **Chromosomal regional view (indicating the *specie*); **(iv) **Gene locus and transcripts view; **(v) **Expression view (profile in different tissues); **(vi) **Probesets table: *Affymetrix *Probesets which map on [gene locus name]; **(vii) **Probes table: *Affymetrix *Probes which map on [gene locus name].

An illustrative **workflow **for the general use of GATExplorer is included in the front page of the website to facilitate a practical guide of the application. The transcript and protein sequences within each gene locus are also provided in a link called "Show SEQUENCES (cDNA)" included within the "Description" of each gene. Some other useful links and tools are included in the "Description" box: one external link to the corresponding gene in *Ensembl *(indicating the ENSG ID); another external link to the corresponding proteins associated to this gene in the *Protein Atlas *database http://www.proteinatlas.org; a tool called "Bookmark GENE" that builds a new box inside the web with direct links to genes selected by the user: bookmarked genes.

The server can be queried using five access-boxes located on the left side which receive the following types of queries: a **keyword **related to any gene locus; a **probe **or **probeset **ID from *Affymetrix*; a **list of probesets **to find corresponding genes; a **sequence **(nucleotide or amino acid) via BLAST; or a range of **chromosomal coordinates**. The usage of each of these access tools is explained in detail in the "Help" section (link on the top right side of the main page).

Accurate graphical representations of genes, transcripts and probes in the "Chromosomal regional view" and the "Gene locus and transcripts view" are achieved using MING (a library for generating *Flash *files), which produces vector drawings in SWF format maintaining the scale of each exon and intron in proportion to their sequence length. Interactive links, gene descriptions, exonic structure and probe positions are included in the drawings. Each microarray probe is identified by its sequence, which is included in the "Probes table" together with its GC content (%). The "Probesets table" and "Probes table" include links ("Download PROBESETS (.txt)", "Download PROBES (.txt)") on the top right to download text files containing the probesets or the probes that map to the selected gene locus. The complete mappings for each microarray platform are included in the PROBE MAPPING section, which is opened in another browser window (link on the top right side of the front page of the website).

The PROBE MAPPING section includes several pages divided into two parts: **(i) **pages providing the complete collection of files that can be downloaded by the user to facilitate the application of the mappings to any particular microarray dataset that is to be analyzed ("Text Files", "R Packages" and "Annotation Files"); **(ii) **pages to explain how the mapping has been performed and provide data to compare the results with other methods previously reported ("Methods", "Statistics", "Comparative Analysis" and "Genomes & Databases VERSION"). Detailed descriptions of the downloadable files included in this section of the database are as follows:

• text files (.txt) with complete unambiguous mapping of the microarray probesets to genes (*probesets2 genes*); • text files (.txt) with complete unambiguous mapping of array probes to genes (*probes2 genes*); • text files (.txt) with complete unambiguous mapping of array probes to transcripts (*probes2transcripts*); • text files (.txt) with complete mapping of microarray probes that are ambiguous because they map on more than one gene locus (*ambigprobes2 genes*).

• R chip definition files (CDFs) with complete unambiguous mapping of microarray probes to genes (*GeneMapper*); • R chip definition files (CDFs) with complete unambiguous mapping of array probes to transcripts (*TranscriptMapper*); • R chip definition files (CDFs) with complete unambiguous mapping of array probes to exons (*ExonMapper*); • R chip definition files (CDFs) with mapping to ncRNAs of the probes that did not map any known protein-coding exon (*ncRNA Mapper*).

• annotation files with information about the mapped entities derived from the *Ensembl *database: genes (ENSGs), transcripts (ENSTs) and exons (ENSEs); • annotation files that include only the selected subset of the protein-coding genes (i.e. the *Ensembl *gene loci, ENSGs, that correspond to mRNAs) or the selected subsets of known microRNAs (i.e. the *Ensembl *gene loci that have been assigned to microRNAs).

The PROBE MAPPING section also presents details regarding the specific "Methods" used, the "Statistics" regarding the mapping to different transcribed entities and a "Comparative Analysis" with other related applications. The "Methods" page provides descriptions and links to the main data sources used in GATExplorer and a graphical schematic view of the pipeline followed to build the web platform, presenting the main steps and procedures applied and the files and packages provided by the server. The "Statistics" page provides the data derived from the sequence mapping of all the oligonucleotide probes from *Affymetrix *expression microarrays into different types of RNAs. Probes are classified as mapping to: protein-coding RNAs (mature mRNAs), non protein-coding RNAs (ncRNAs) or unassigned to any known RNA (NA). Probes that only map to introns were classified as mapping to putative ncRNAs. The percentage of probes mapping to each class is provided for four types of widely used human expression microarrays platforms. The page also provides statistics on the number and percentages of transcripts and genes mapped by the probes in each *Affymetrix *expression microarray (for human *Homo sapiens*, mouse *Mus musculus *and rat *Rattus norvegicus*); and the number and percentages of probes that map to transcripts and genes with respect to the total in each array. The "Comparative Analysis" page includes a comparison of GATExplorer with other related applications that have been previously published. The page examines four studies that have undertaken an alternative mapping of probes to genes for *Affymetrix *microarrays. Some of these re-mapping approaches and tools are limited to a subset of microarray platforms or do not apply to whole-transcript expression microarrays (i.e. Gene 1.0 and Exon 1.0). Among the previous studies, none present mapping to intronic regions or ncRNAs.

**Figure 1:**
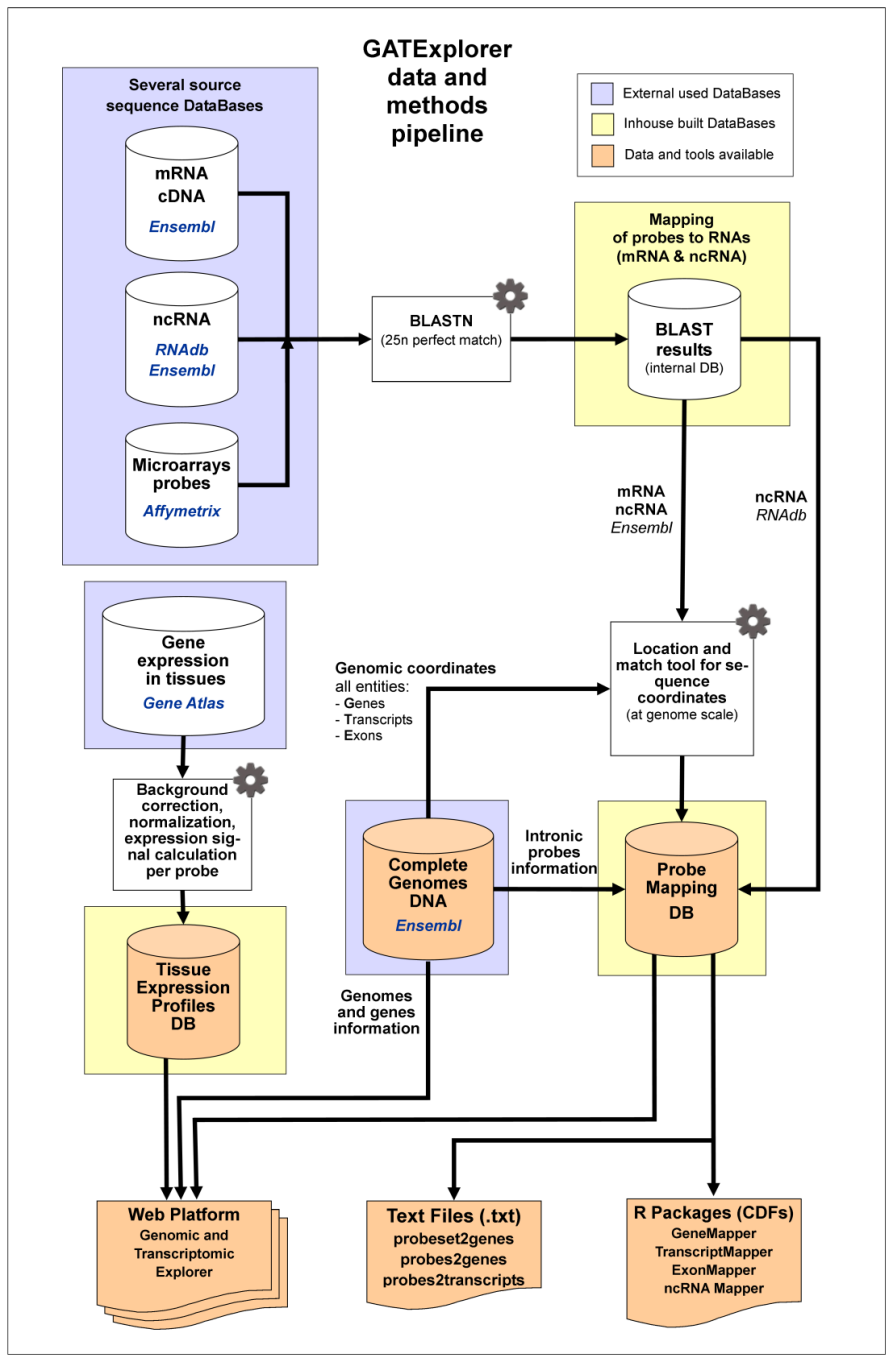
**Graphical view of the pipeline followed to build the GATExplorer application, showing the data sources integrated (*Ensembl*, *RNAdb*, *Affymetrix *and *GeneAtlas *-i.e. the four external databases marked in blue square frames-) and a schematic view of the main methods applied at each step -marked with grey gears**. As a result, the outputs from the pipeline provide the web platform, which encloses several inhouse built databases -marked in **yellow **square frames- and several data and tools -marked in **orange**-, including the files (Text files) and packages (R CDFs) with the *de novo *mapping of the array probes to the transcriptome of the corresponding organism (human, mouse or rat). The application also provides the genomic coordinates to each locus (including mapping on exons, introns and along exon-exon junctions) and identification of any intersecting genes, transcripts, exons and ncRNAs.

Figure 1 presents a graphical view of the main data sources and methods included in GATExplorer described above. The graph shows the pipeline followed to build the database and web platform. The probe mapping files (Text files) and packages (R CDFs) are freely provided as part of the repository to allow researchers to use the microarray probe remapping data for their own expression analyses.

Each gene can be queried to find detailed information on the mapping of probes to their corresponding locus, transcripts and exons. When a gene loci is shown in the associated GATExplorer web page, the "Gene locus and transcripts view" presents all the probes that map to such loci for each of the *Affymetrix *microarrays. As mentioned above, the information about such probes indicating whether they are ambiguous (i.e. multi-mapping) or not is included in the table called "Probes table". After mapping, each probe is designated with a COLOR CODE (green, yellow, red and black) to indicate whether it is ambiguous or not (see HELP section). As a result, all ambiguous probes that can cross-hybridize with several biological entities (i.e. >1 gene or transcript or exon) are identified. The probes that are transcript-specific or exon-specific are provided in a link ("Probes ... specific" link) to another page that includes the list of corresponding *Ensembl *ENST or ENSE IDs. The "Expression view" provides the expression profile of the queried gene in a set of different organs, tissues or cell types (Su *et al*. 2004 dataset [6]) and shows the expression signal per probe for each of the probes assigned to this gene locus or the global expression signal as log2 of the mean of all probes.

We provide an **example **to facilitate the use of the described views and tools included in GATExplorer: human gene MEST (mesoderm-specific transcript homolog genes, *Ensembl *ENSG00000106484). This gene is located on chromosome 7 and its locus is 20.08 Kbp long. It has 4 transcripts and 16 exons. It is mapped by 175 distinct *Affymetrix *probes, which are included amonst 9 different microarray platforms. In the case of array *HGU133 plus 2*, 11 probes map to this gene, which correspond to *Affymetrix *probeset 202016_at. This probeset does not include any "transcript-specific probe" because all probes map to the 4 known transcripts. For this gene the highest expression measured corresponds to bone marrow samples.

## Utility and discussion

### Mapping genes, transcripts and exons: coverage and efficiency

Accurate expression determination requires that microarray probes have minimal cross-hybridization with other genes or other transcribed entities. The GATExplorer database includes detailed information regarding the *coverage *and *efficiency *of the probe mapping (Tables 1, 2). *Coverage *is defined as the proportion (i.e. %) of gene loci or transcripts from the total genes/transcripts of the *Ensembl *genomes (human, mouse or rat) that are mapped by the probes of a given microarray. *Efficiency *is defined as the proportion (%) of probes from the total probes of a given microarray that map to *Ensembl *genes or transcripts. The term "unique mapped" refers to those gene loci or transcripts that are targeted by a set of probes of a given microarray that do not cross-hybridize (i.e. map unambiguously) with any other known gene loci or transcript.

**Table 1:** *Coverage *of the probe mapping.

	**Transcripts**				**Gene Loci**				**TOTAL N of Transcripts**	**TOTAL N of Gene Loci**
			
	**Unique mapped**		**All mapped**		**Unique mapped**		**All mapped**			
			
	**N transcripts**	**%**	**N transcripts**	**%**	**N gene loci**	**%**	**N gene loci**	**%**		
	
**Microarray**										
HG U133A	7198	**13,57%**	31219	**58,88%**	12218	**44,95%**	15048	**55,36%**	53024	27184
HG U133 Plus 2.0	11755	**22,17%**	42819	**80,75%**	17710	**65,15%**	20764	**76,38%**	53024	27184
Human Gene 1.0	19947	**37,62%**	48169	**90,84%**	19923	**73,29%**	22446	**82,57%**	53024	27184
Human Exon 1.0	28024	**52,85%**	51851	**97,79%**	23967	**88,17%**	26047	**95,82%**	53024	27184

*Coverage *of the probe mapping for four human *Affymetrix *microarray models: U133A, U133 Plus 2.0, Gene 1.0 and Exon 1.0. *Coverage *is defined as the proportion of gene loci or transcripts from the total genes/transcripts of the *Ensembl *human genome that are mapped by the probes of a given microarray. The term "unique mapped" indicates the gene loci or transcripts mapped by a unique set of probes of a given array that do not cross-hybridize with any other known gene loci or transcript (i.e. such probes are not ambiguous). N corresponds to number of gene loci or transcripts.

**Table 2:** *Efficiency *of the probe mapping.

	**Transcripts**				**Gene Loci**				**TOTAL N of probes mapped**	**TOTAL N of probes in the microarray**	**Mapping efficiency**
				
	**1**		**>1**		**1**		**>1**				
				
	**N probes**	**%**	**N probes**	**%**	**N probes**	**%**	**N probes**	**%**			
	
**Microarray**											
HG U133A	85075	**44,60%**	105677	**55,40%**	180188	**94,46%**	10564	**5,54%**	190752	241898	**78,86%**
HG U133 Plus 2.0	150060	**47,74%**	164260	**52,26%**	299482	**95,28%**	14838	**4,72%**	314320	594532	**52,87%**
Human Gene 1.0	387229	**54,45%**	323912	**45,55%**	658258	**92,56%**	52883	**7,44%**	711141	804372	**88,41%**
Human Exon 1.0	614549	**45,75%**	728669	**54,25%**	1263553	**94,07%**	79665	**5,93%**	1343218	5270588	**25,49%**

*Efficiency *of the probe mapping for four human *Affymetrix *microarray models: U133A, U133 Plus 2.0, Gene 1.0 and Exon 1.0. *Efficiency *is defined as the proportion of probes from the total probes of a given microarray that map to *Ensembl *human genes or transcripts. N corresponds to number of probes.

The quantity and percentage of human gene loci and transcripts targeted by the most widely used human *Affymetrix *expression microarrays based on 3' expression (U133A and U133 Plus 2.0) and the new all-exon arrays (Gene 1.0 and Exon 1.0) is summarized in Table 1. The data shows that the Gene 1.0 and Exon 1.0 arrays achieve the highest coverage over gene loci: 82.57% and 95.82%, respectively (mapping to a total of 27184 human genes, obtained from genome assembly *Ensembl v53 NCBI36*). Such coverage (which depends on the quality of the genome annotation) has improved with respect to previous array models; for example, U133A shows 55.36% coverage of the current *Ensembl *genes. The transcript coverage also improves in the newer models (mapping to a total of 53024 human transcripts, obtained from the same genome assembly). However, the coverage decreases when "unique mapped" genes or transcripts are considered. For example, in the case of the human Gene 1.0 array, 73.29% of the genes are mapped by unique sets of probes. In any case, the overall coverage to measure expression from most human gene loci has improved by 27%, from U133A (55%) to Gene 1.0 (82%).

With respect to the efficiency of the probe mapping, Table 2 presents the number and percentage of distinct probes in each microarray (i.e. probes of distinct sequence) that map to one or more transcripts or gene loci, for the most commonly used human microarray models. Therefore, the columns with >1 include the probes that map to more than one transcript or gene locus. Those probes that map to several transcripts or loci, can be considered "ambiguous" probes. The figures show that the best mapping efficiency (88.41%) is obtained with the Gene 1.0 array. For the U133A array, 78.86% of the probes map to known gene loci of the current human genome version (*Ensembl v53 NCBI36*). The mapping efficiency decreases even further, to 74.5% (180188/241898), when only probes that hybridize to one gene locus are considered (e.g. 180188 probes for U133A). Therefore, probe mappings to human cDNA show that a significant portion (5.54% for U133A and 7.44% for Gene 1.0) hybridize "ambiguously" to more than one gene locus. A larger percentage of probes (55.40% for U133A and 45.55% for Gene 1.0) can hybridize to more than one transcript. Therefore, only a certain percentage of probes can be regarded as gene-specific or transcript-specific. As a general conclusion, these calculations indicate that a significant proportion of probes (about 20 to 25% when mapping genes with U133A) can produce noise using standard expression signal calculations based on the probesets assigned by *Affymetrix*.

**Figure 2:**
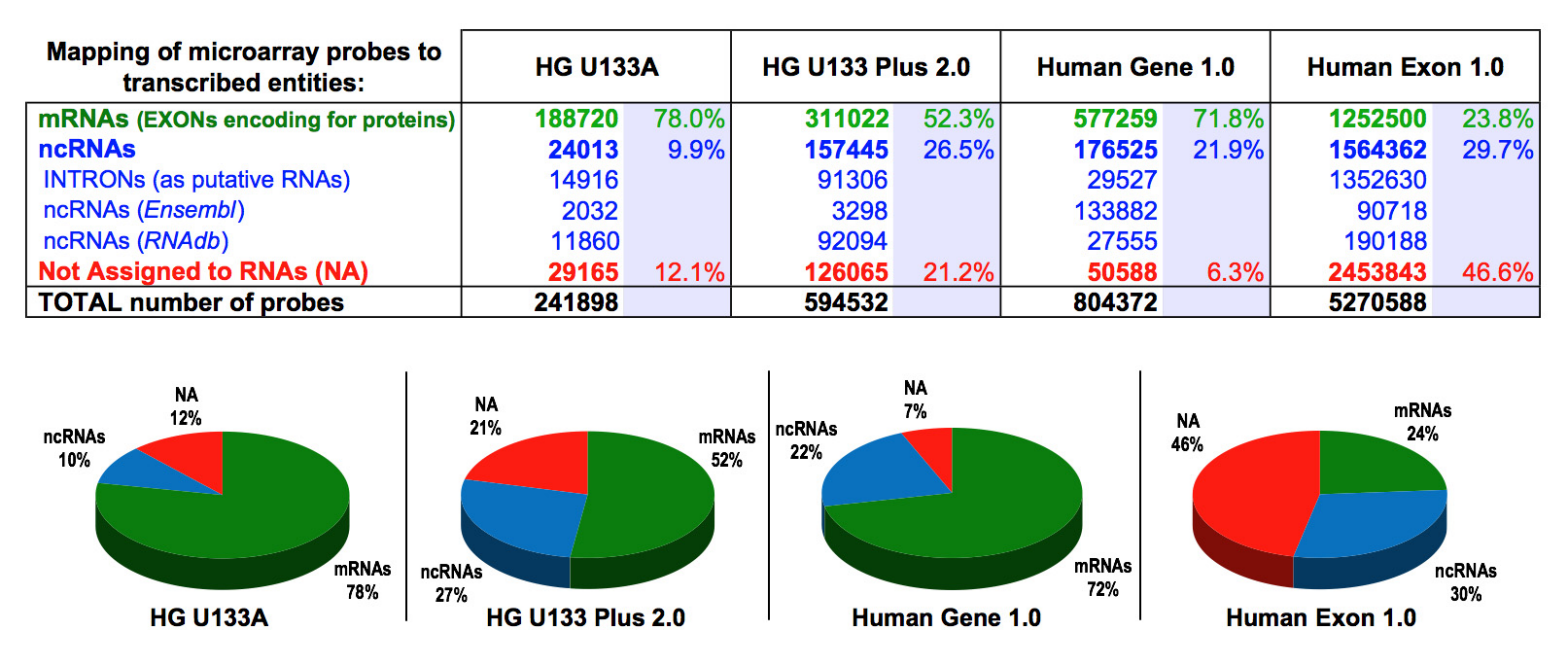
**Table and pie graphs summarizing the probe mapping from four widely used human *Affymetrix *microarray models (U133A, U133 Plus 2.0, Gene 1.0 and Exon 1.0) to different transcribed entities**. The figure includes information about the mapping and assignment of probes considering tree types: **(i) green**, probes mapped to translated genes (mRNAs, i.e. exons encoding for proteins); **(ii) blue**, probes mapped to ncRNAs (derived from *RNAdb *and from genes labeled as non-protein-coding in *Ensembl*); **(iii) red**, probes not mapped to any known RNA (NA, not assigned).

The described problem is also present in the new Exon 1.0 arrays, which show the lowest efficiency with only 25.5% of the probes mapping to known genes. This apparently low efficiency is not contradictory with a newly manufactured array, because the Exon 1.0 arrays have been designed with a different goal to previous gene expression arrays, which was not just to cover known genes, but to be able to distinguish the expression of each exon in a given locus. To achieve this goal, the array includes a complex collection of exon probes that correspond to five types of probesets based on different degrees of evidence: core, extended, full, ambiguous and free. Descriptions of each of these probesets can be found in the *Affymetrix *white paper on the Exon arrays (called exon_array_design_technote.pdf), which is available from the *Affymetrix *website http://www.affymetrix.com/support/help/exon_glossary. The most important probesets correspond to the "core" type, which are the ones supported by the most reliable evidence from *RefSeq *and full-length mRNA *GenBank *records containing complete CDS information (see exon_array_design_technote.pdf). Recent analytical tools for the Exon 1.0 arrays recommend use of just the "core" set [7]. In the case of human Exon 1.0, the "core" set is composed of 1,082,385 probes http://www.aroma-project.org/chipTypes/ and these probes are mostly included in the set of 1,252,500 probes that GATExplorer assigns to mRNAs exons for this array (see Figure 2). These numbers show that the probe remapping data used in GATExplorer allows the use of a larger set of probes than the "core" set described by *Affymetrix*.

The analysis of coverage and efficiency presented in Tables 1 and 2 should be considered together with the analysis presented in Figure 2, which includes information about the mapping and assignment of all probes from different arrays to protein-coding genes and to ncRNAs (derived from *Ensembl *and from *RNAdb*). The first part, corresponding to the assignment to mRNAs, is marked in green in the pie graphs in Figure 2. These green sectors indicate the proportion of probes that map to known genes, which are large in the case of expression arrays specially designed to measure genes, as human U133A and Gene 1.0 with green sectors of 78.0% and 71.8%, respectively. The blue sector corresponds to ncRNAs, which is exclusively provided by the GATExplorer database. In this sector, we have included those probes that map within "introns" because these probes may measure signal from putative exons included in alternative mRNAs. Indeed, the proportion of probes mapping in such putative or hypothetical exons is comparatively large in the exon arrays (1,352,630 probes of human Exon 1.0), showing that this array is designed to measure many alternative mRNAs. As indicated above, the exon arrays are not only designed to detect mRNAs and for this reason they include many other probes apparently not assigned to any RNA (NA, red sector in pie graphs). Some of these probes correspond to oligos designed for the exon boundaries or for the UTR 3' and 5' borders. Many of these probes map to non-genic regions with little evidence to support their transcription. However, it has been reported that the UTR regions of many human and mouse genes are not well defined, so expression of some of these probes would be anticipated [8]. Nevertheless most of the probes on the human Exon 1.0 array are outside of the "core" reliable set. Complete information and statistics regarding the coverage and efficiency of each microarray platform from human, mouse and rat are included in the GATExplorer database in the PROBE MAPPING section.

### Comparison of the use of genes versus probesets in expression calculations

**Figure 3:**
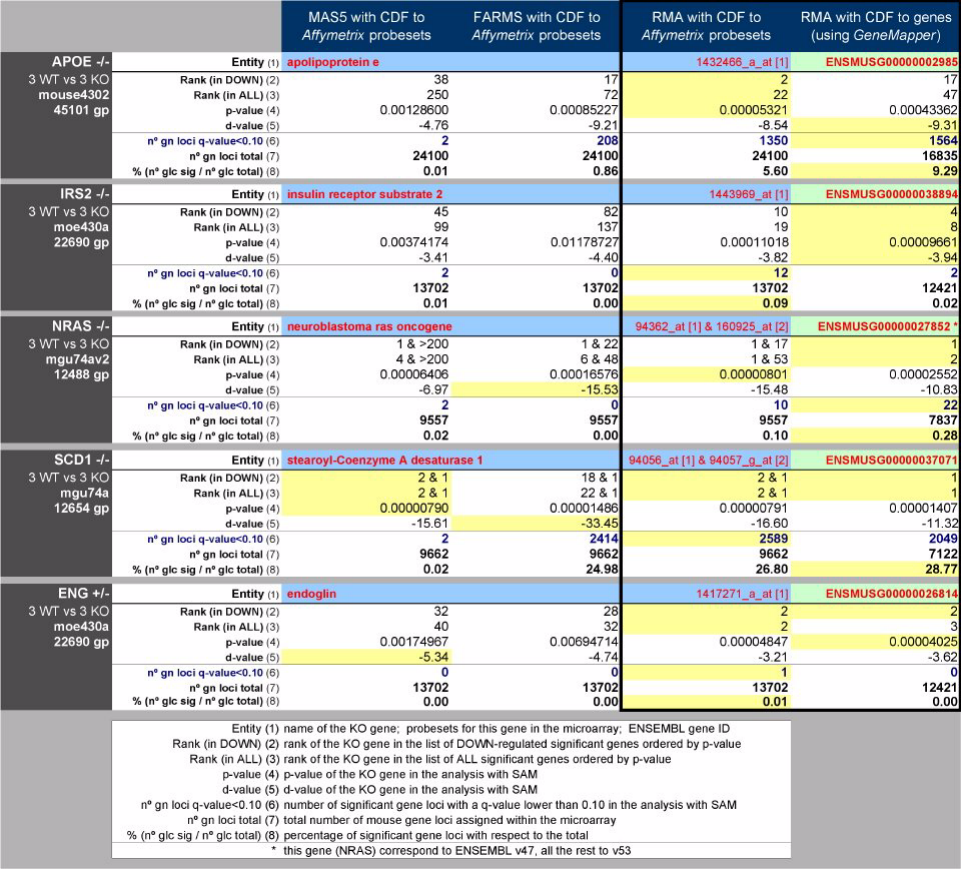
**Comparison of the differential expression calculated by the SAM algorithm for a series of data of mouse microarrays (five sets of six samples) analyzed using three different expression signal calculation algorithms (MAS5.0, FARMS and RMA) with standard CDFs to "probesets" or using RMA with CDFs to "genes" (*GeneMapper *CDFs)**. Each set includes three biological replicates of knock-out (KO) mice for a specific gene compared to three replicates of the corresponding wild-type (WT) mice. The gene KOs are: APOE-/-, IRS2-/-, NRAS-/-, SCD1-/- and ENG+/-. The full name of these genes, the *Ensembl *ID number (ENSG) and the probesets assigned by *Affymetrix *are indicated in the top line of each set, labelled Entry **(1)**. The table shows the numbers for the statistical parameters calculated in the comparison, which are: **(2) **rank of the KO gene across down-regulated genes; **(3) **rank of the KO gene across all genes; **(4) **p-value from SAM for the KO gene; **(5) **d-value from SAM for the KO gene; **(6) **number of significant gene loci with q-value < 0.10 (this calculation was performed such that all probesets were assigned to specific genes following the *Affymetrix *assignment or the *GeneMapper *assignment; therefore the methods are comparable since the number of gene loci indicated are the fraction of total mouse genes assigned); **(7) **total number of mouse gene loci assigned within the microarray; **(8) **percentage of significant gene loci with respect to the total. Yellow background indicates the top values for each statistical parameter calculated with each of the four procedures used. The comparison that includes the identical methods for expression calculation (**RMA**) and for differential expression (**SAM**) changing only the CDFs is presented in the last two columns, framed with a black line.

In the expression profiles provided by the *GEO *database and in the many associated publications http://www.ncbi.nlm.nih.gov/geo, the most common approach to calculate gene expression signals is using the *Affymetrix *probesets as direct synonyms of genes. The underlying assumptions carry considerable risks and there are few comparative expression studies that investigate the value of using up-to-date mapping of probes to genes, although it has been reported that this approach improves the precision and accuracy of microarrays [9]. Therefore, to investigate how the application of the remapping may affect the expression data, we present in Figure 3 the results of a comparative study of several microarray datasets that were analyzed either using the standard Chip Definition Files (CDFs) to "probeset" or using the new Chip Definition Files (CDFs) that include the "gene-specific" remapping and assignment, provided by GATExplorer. These analyses are performed using first three different expression signal calculation algorithms (**MAS5.0**, **FARMS **and **RMA**) with CDFs to "probesets" and then using **RMA **with CDFs to "genes" (i.e. using the *GeneMapper *packages) [10-12]. These three algorithms are well-known (*Affycomp *website affycomp.biostat.jhsph.edu) and **RMA **is nowadays the most widely used to calculate microarray gene expression signals [13,14]. Following the application of the expression calculation algorithms with different CDFs, a common robust algorithm for differential expression called **SAM **was applied to all the data [15]. All the analyses were performed using R and the *BioConductor *packages (see website: http://www.bioconductor.org/).

The datasets are a collection of mouse microarray experiments (including four different *Affymetrix *platforms) corresponding to five sets of six samples. Each set includes three biological replicates of knock-out (KO) mice for a specific gene that are compared to three biological replicates of the corresponding wild-type (WT) mice. The five gene KOs are: APOE-/-, IRS2-/-, NRAS-/-, SCD1-/- and ENG+/-. The full name of these genes, the *Ensembl *ID number (ENSG) and the probesets assigned by *Affymetrix *are indicated in Figure 3. Three genes have a unique *Affymetrix *probeset (APOE, IRS2 and ENG) and two genes have two probesets (NRAS and SCD1).

The main feature to be evaluated in the comparison is: how the mapping with the CDFs to "probesets" and the mapping with the CDFs to "genes" affect the detection of the KO genes. In optimum conditions, the gene that is not present in the KO mice should suffer one of the most dramatic differences when compared with the WT and show a significant "repression" or "down-regulation". *A priori *we do not know how many other genes can be affected by the KO gene and we do not know the overall biological/functional signature associated to each KO gene. For this reason, we do not assume that the KO gene will always be the most repressed.

The data and statistical parameters calculated in the comparison, shown in Figure 3, are: **(1) **full name of the gene, corresponding probesets assigned by *Affymetrix *and *Ensembl *ENSG ID number; **(2) **rank of the KO gene across down-regulated genes; **(3) **rank of the KO gene across all genes; **(4) **p-value from **SAM **for the KO gene; **(5) **d-value from **SAM **for the KO gene; **(6) **number of significant genes with q-value < 0.10 (using the assignment of probesets to genes provided either by *Affymetrix *or by *GeneMapper*); **(7) **total number of mouse genes assigned within the microarray; **(8) **percentage of significant genes with respect to the total.

The highest ranked statistical value among the four comparisons is highlighted in yellow (although, it is important to note that the highest statistical rank does not imply the most biologically relevant change). In four out of five cases (IRS2, NRAS, SCD1 and ENG) the newly calculated gene mapping provides a better rank than the standard mapping, according to the statistical significance of the differential expression of the KO gene. The number of genes with *q-value *< 0.10 (which indicates the extension of the significant change) was the largest with the newly calculated gene mapping for two KO genes: APOE, 9.29% changed genes; NRAS, 0.28% changed genes. Finally, the *p-value *of the statistical test was lowest with the new mapping for KO genes ENG and IRS2. The results consistently indicate that the method using CDFs with the new remapping to "genes" provides at least as significant changes as the best of the three methods based on *Affymetrix *"probesets" CDFs.

We emphasize that the purpose of these analyses is not to propose a new algorithm, but rather to determine in a comparative approach whether the array probe remappings provide results that are at least of equal quality to the original probesets. A complete evaluation of the methods will need a deep biological and functional analysis of the results that goes beyond the scope of this paper. To facilitate further analysis and independent comparison, we provide in the website the raw datasets (CEL files) corresponding to the results presented in Figure 3. Moreover, APOE, NRAS and SCD1 microarray samples can be downloaded in GEO database: GSE2372, GSE14829 and GSE2926, respectively http://www.ncbi.nlm.nih.gov/geo.

### Remapping expression probes to ncRNAs

As indicated above, the proportion of probes on the human arrays that map to genes was 78.9% for U133A, 52.9% for U133 Plus 2.0 and 25.5% for Exon 1.0. This efficiency is fractionally lower when only "protein-coding gene loci" (i.e. loci that encode mRNAs translated to proteins) are considered: 78.0% for U133A, 52.3% for U133 Plus 2.0 and 23.8% for Exon 1.0 arrays. This shows that a large fraction of probes within these microarrays do not map to any known protein-coding RNA (i.e. mRNA). Therefore, we performed a remapping of those probes not assigned to mRNAs to a database of ncRNA sequences (*RNAdb v.1 *from 2009) [4]. These ncRNAs belong to the mRNA-like class of long ncRNAs. These were predominantly identified in cDNA libraries, such as those used in the *FANTOM3 *and *H-Invitational *datasets [1,16]. Because cDNA library generation typically involves poly-dT priming, such cDNA sequences largely arise from polyadenylated transcripts. However, due to the possibility of internal priming from polyA-rich tracts, some non-polyadenylated transcripts may also be present. Nevertheless, such transcripts are represented in any gene expression study that employs a microarray protocol that selectively amplifies polyadenylated transcripts by poly-dT priming. The new generation *Affymetrix *microarrays Gene 1.0 and Exon 1.0 use WT random primed amplification, which does not necessitate the presence of a poly-A tail. This type of microarrays can detect many more ncRNAs.

The results of the remapping of array probes to ncRNAs showed that 29.7% of the probes from human Exon 1.0 and 26.5% of the probes from U133 Plus 2.0 map to ncRNAs. A summary of this remapping is presented in Figure 2, which shows the percentages of probes that map to ncRNAs, combining both the information from *RNAdb *and from genes labeled as non-protein-coding in *Ensembl*. These data also include the probes that only map within introns.

Expression of ncRNAs is becoming of increasing interest due to the accumulating evidence showing that ncRNAs are biologically relevant. A key question in investigating ncRNA function is determining whether ncRNAs produce distinct expression signals or just reflect background "noise". To check the variability and detectability of changes in expression provided by the ncRNAs, we selected the 92,094 probes from the U133 Plus 2.0 array that map to ncRNAs according to our mapping to *RNAdb *(see Figure 2). This set of probes is provided in the CDF package "ncRNA Mapper" (file ncrnamapperhgu133plus2cdf_1.0). This CDF file was applied to a microarray dataset obtained from GEO (GSE3526), which includes 353 arrays corresponding to samples from 65 different normal human tissues. The expression of the ncRNAs assigned by the CDF package was calculated using the **RMA **algorithm. Following the calculation of the expression signals, we determined the number of ncRNAs showing significant differential expression by performing a statistical analysis of variance using an *anova *test (function *aov *from *stats *R package). This analysis indicated that 70.5% of the assigned ncRNAs showed differential expression with p-values < 0.01 (p-values corrected by *Bonferroni *method). This means that 4,274 ncRNAs (out of 6,062) changed in all replicates in at least one set of tissues. These results reveal the importance of considering expression signals coming from ncRNAs in transcriptomic studies. Moreover, there is an increasing number of reports showing the biological importance of new transcribed entities that do not encode for proteins, and demonstrate that many play important and diverse roles in cellular function [17,18].

## Conclusions

### Beyond the "gene" in microarray studies

Genome-wide expression studies have developed exponentially in recent years due to the use of microarray technology [5]. Presently, the most reproducible and widely used microarrays are high-density oligonucleotide microarrays, which feature synthetic oligos based on cDNA and EST sequences. New high-throughput RNA-sequencing will become an excellent complement to microarray datasets, providing highly detailed information about all transcribed entities [19]. However, due to the large number of studies performed with expression microarrays (both past and present) it remains worthwhile to improve the manner by which these data are analyzed. The specific assignment of array probes to current gene annotations, transcripts and exons and the provision of tools to visualize array expression signals in an updated genomic context represent a significant enhancement to currently available methods. With the continued erosion of traditional definitions of "gene" being exposed through transcriptomic sequence data [20,21], as well as the increasing importance of ncRNAs in understanding disease and development [17], the data and analytical techniques enabled by GATExplorer comprise an important aspect for the meaningful interpretation of microarray expression information and its integration within the transcriptome.

The first attempt to provide alternative mapping of *Affymetrix *microarray probes to the latest versions of human genes was reported by Gautier *et al*. in 2004 [22]. Since this report, several studies have been published providing redefinitions of *Affymetrix *microarray probe and probesets to genes and transcripts, including tools to use such redefinitions [22-29]. Dai *et al*. developed probably the most comprehensive mapping of microarray probes from several species [23]. Despite the reannotation of *Affymetrix *microarray probes and probesets to genes and transcripts having been reported previously, GATExplorer is the first system that integrates mapping of probes (including maps to ncRNAs) with simple genomic contextual views, as well as expression signals at probe level. A study and comparison of the characteristics of five major applications that have undertaken an alternative mapping of probes to genes for *Affymetrix *microarrays can be seen in the "Comparative Analysis" page of the PROBE MAPPING section of GATExplorer (the comparison corresponds to references [22-24,28] and this work).

Regarding the visualization of the probes in a genomic context, current genome browsers (such as the *UCSC *browser: http://genome.ucsc.edu/, and *Ensembl *browser: http://www.ensembl.org) incorporate large amounts of data with complex genome-wide information, including location of the probesets from microarrays. Other web sites, like *X:map*, provide specific annotation and visualization of *Affymetrix *exon arrays probesets and probes within the genome structure [30]. *Exon Array Analyzer *is a web tool that allows analysis of exon arrays to detect differentially expressed exons and places the probes within the corresponding genes [31]. The open-source software *BioConductor *(http://www.bioconductor.org/), includes a package called *GenomeGraphs *to plot genomic information from *Ensembl*, which uses *biomaRt *to query the genomes database and transform gene/transcript structures to graphical views, with the possibility of including probes from exon arrays. *Affymetrix *has also developed an application for visualization and exploration of genes, genomes and genome-scale data sets, called *Integrated Genome Browser *(IGB) that uses the *GenoViz *and *Genometry *software (http://genoviz.sourceforge.net/). Although these applications are useful, they fulfil demands that differ to that of GATExplorer. The importance of the work presented here lies in the demonstration of the large proportion of probe targets that microarrays detect which most researchers do not consider in an expression experiment, and to allow them to use the expression signals arising from non-coding RNAs and hypothetical exons.

In conclusion, GATExplorer is an integrated database and web platform that is useful to visualize, analyze and explore the increasing complexity of eukaryotic transcriptomes (human, mouse and rat), which includes microarray probes mapping to gene loci, transcripts and exons (even exon-exon junctions), as well as introns and ncRNAs.

## Availability and requirements

The database is available at http://bioinfow.dep.usal.es/xgate/. GATExplorer is open access and the website makes available all the files and packages described here. The website also includes a "Help" section to facilitate the use of the application.

## Authors' contributions

AR is the main developer and current system manager of the database, who has carried out most of the programming code including several interactive tools and the construction of the relational database. CF has helped in the software development and in the improvement of the web pages. She has also contributed in the active discussion and correction of the manuscript. MED has provided updated ncRNAs databases and has actively contributed to the improvement of the web pages of the database and to the writing of the manuscript. JR has made a major contribution to conception and design of the database and several of the tools included, he is the corresponding author who carried out the writing of the manuscript and revising its intellectual content. All authors read and approved the final manuscript.
